# Trabectedin for inoperable or recurrent soft tissue sarcoma in adult patients: a retrospective cohort study

**DOI:** 10.1186/s12885-016-2054-2

**Published:** 2016-01-19

**Authors:** Fernando A. Angarita, Amanda J. Cannell, Albiruni R. Abdul Razak, Brendan C. Dickson, Martin E. Blackstein

**Affiliations:** Division of General Surgery, Department of Surgery, University of Toronto, Toronto, ON Canada; Department of Medical Oncology, Mount Sinai Hospital, Toronto, ON Canada; Department of Medical Oncology, Princess Margaret Cancer Centre, University Health Network, Toronto, ON Canada; Department of Medicine, University of Toronto, Toronto, ON Canada; Department of Pathology and Laboratory Medicine, Mount Sinai Hospital, Toronto, ON Canada; Department of Laboratory Medicine and Pathobiology, University of Toronto, Toronto, ON Canada

## Abstract

**Background:**

Trabectedin is an antineoplastic agent used for patients with soft tissue sarcoma (STS) who fail standard-of-care treatment. Real-world data of its performance is scarce. This study evaluates the safety and effectiveness of trabectedin for patients with advanced STS who were treated at a high-volume sarcoma center.

**Methods:**

A retrospective chart review was performed on 77 patients treated with trabectedin (24 h infusion q3w) between 01/2005 and 05/2014. Data regarding safety, objective radiological response, progression-free and overall survival were analyzed.

**Results:**

Median age at treatment onset was 52y [interquartile range (IQR): 45-61y]. Tumors included leiomyosarcoma (41.6 %), liposarcoma (18.2 %), and synovial sarcoma (13 %). Trabectedin was provided as ≥ third-line chemotherapy in 71.4 %. Median number of cycles was 2 (range: 1–17). Dose reduction and treatment delays occurred in 19.5 and 40.3 %, respectively. Toxicities occurred in 78 %, primarily for neutropenia or elevated liver enzymes. Two patients died secondary to trabectedin-induced rhabdomyolysis. Treatment was discontinued because of disease progression (84.7 %), toxicity (10 %), and patient preference (5 %). Partial response or stable disease occurred in 14.1 and 33.8 %, respectively, while 52.1 % developed progressive disease. Median progression-free survival was 1.3 m (IQR: 0.7–3.5 m) and was significantly higher in patients lacking severe toxicities or progressive disease. Median overall survival was 6.7 m (IQR: 2.3–12.7 m) and was significantly higher in patients with leiomyosarcoma or liposarcoma relative to other histologies.

**Conclusions:**

Trabectedin has an acceptable safety profile as an anti-tumor agent. Our data further suggest there may be some benefit in using trabectedin particularly in patients with leiomyo- or liposarcoma who failed standard-of-care agents.

## Background

Soft tissue sarcomas (STS) are rare solid cancers of mesenchymal cell origin accounting for <1 % of adult cancers [1]. Considerable heterogeneity exists with over 50 histologic types of STS, each with distinct clinical behaviour. Despite this heterogeneity, adult patients with advanced STS are generally treated similarly with palliative-intent chemotherapy. Few agents have known anti-tumor activity in advanced STS, but generally provide limited benefits in survival outcome. Guidelines recommend anthracycline-based chemotherapy as first-line treatment for most advanced STS [2, 3] with a 26 % response rate [4]. Another drug used as first-line treatment is ifosfamide, which with or without doxorubicin offers a response rate of ~25 % [5]. Treatment options for patients who fail first-line treatment include gemcitabine/docetaxel [3], but additional agents are scarce.

Trabectedin is the synthetic version of an anti-cancer alkaloid agent originally isolated from the Caribbean sea squirt *Ecteinascidia turbinate* [6]. Trabectedin covalently binds to the DNA minor groove at guanine nucleotides of specific sequences to inhibit gene activation and repair mechanisms and induce lethal DNA double-strand breaks that ultimately lead to cell cycle arrest [7]. Additionally, recent studies suggest pleiotropic properties [8–11]. Trabectedin selectively targets macrophages and down-regulates the production of pro-inflammatory mediators, changing the tumor microenvironment and contributing to anti-cancer activity [8–10]. Trabectedin also promotes cancer cell differentiation, specifically in myxoid liposarcoma by modulating the transcription of genes crucial for adipocytic differentiation [11].

Trabectedin has shown efficacy as salvage chemotherapy in patients with advanced STS in three phase II trials [12–14], chemotherapy-naive patients with unresectable advanced disease [15], and in compassionate use programs [16–18]. An open-label, randomized, phase II study evaluated two regimens in patients with unresectable advanced or metastatic liposarcoma or leiomyosarcoma [19]. This study established that trabectedin (1.5 mg/m^2^ given as a 24-h intravenous infusion q3w) provided significantly better disease control over weekly 0.58 mg/m^2^ by improving time to progression (TTP) and progression-free survival (PFS). In 2007, based on these results, trabectedin was approved in several countries for use in STS patients who fail standard treatments or are unsuited to receive first-line agents [20, 21].

The effectiveness and safety profile of trabectedin in the aforementioned studies may differ from that of real clinical settings as patients typically go through rigorous enrolment processes before entering clinical trials. Institutional case series provide a suitable means to obtain real-world data. Therefore, our study aimed to evaluate the safety and effectiveness of trabectedin in patients with inoperable or recurrent STS treated at a high-volume academic sarcoma center in North America.

## Methods

### Study design

Research ethics board approval at Mount Sinai Hospital (MSH), Toronto, ON, Canada was obtained in order to identify patients treated with trabectedin from the University of Toronto Sarcoma Group’s medical oncology and pharmacology database. Informed consent was obtained in order to include patients into the database. A retrospective chart review was performed from medical records of patients who initiated treatment between 01/01/2005 and 05/30/2014. Inclusion criteria included ≥18 years old (y); histologically confirmed STS; patients with locally advanced, metastatic, inoperable, recurrent or disease progression after first-line treatment; and at least one treatment cycle. Patients with gastrointestinal stromal tumors were excluded.

### Data collection

Data was extracted by one author (FAA) and 10 % of data was independently corroborated by two additional authors (AJC and MEB). Extracted data included patient demographics and medical history, STS details, pre-trabectedin treatment information, trabectedin information, post-trabectedin treatment information and follow-up information.

### Clinical practice

The University of Toronto Sarcoma Group treats patients at both Mount Sinai Hospital and Princess Margaret Cancer Centre (PM), two high-volume adult sarcoma centres for the province of Ontario. Tumor specimens were classified according to the World Health Organization (WHO) system by an expert soft tissue pathologist (BCD). Patients are generally referred from regional health centres for multidisciplinary management.

To be eligible for treatment patients had to meet the following criteria: ≥18y, biopsy-proven STS, documented unresectable advanced or metastatic tumor, either failure or intolerance to doxorubicin and/or ifosfamide, currently not receiving anti-cancer treatment, Eastern Cooperative Oncology Group (ECOG) performance status ≤1; adequate bone marrow reserve (neutrophils >1500/mm^3^ and platelets >100,000/mm^3^); adequate renal function (serum creatinine <120 μmol/L or calculated creatinine clearance by Cockroft method >60 mL/min); and adequate hepatic function (bilirubin >30 μmol/L, aspartate aminotransferase (AST) and alanine transaminase (ALT) T <1.5U/L or <2.5U/L if liver metastases, alkaline phosphatase (ALP) <2.5U/L and albumin >25 g/L). Contraindications included known history of hypersensitivity to trabectedin or its components, active serious and/or uncontrolled infection, left ventricular ejection fraction below lower normal limit, concomitant live vaccines,  creatine kinase (CK)  > 2.5x upper normal limit, elevated bilirubin and breast feeding.

Trabectedin was generally given at the recommended starting dose (1.5 mg/m^2^) as a 24-h continuous intravenous infusion q3w. Trabectedin was administered via a portable infusion pump that enabled outpatient treatment. Before each cycle patients were assessed to confirm adequate renal, hepatic and bone marrow reserve function as well as overall performance status. Anti-emetic prophylaxis with corticosteroids (20 mg of dexamethasone intravenously administered 30 min pre-trabectedin) was provided. Dose reductions (20 % intervals) were made in the event of toxicities occurring during the previous cycle. Once the toxicity resolved the dose was readjusted at the discretion of the medical oncologist. Dose adaptations were similar to those applied in previously published protocols [12, 14]. There were no pre-defined limits to the number of cycles therefore patients with non-progressive disease and no adverse events continued receiving treatment until progression, grade 4 toxicities, and/or patient preference. Response to treatment was assessed every two cycles by CT scans, which were reviewed by the treating medical oncologist.

### Classifications

Toxicity was retrospectively assessed using the Common Terminology Criteria for Adverse Events (CTCAE) v4.03 classification [22]. Because of the study’s retrospective nature, only hematological and biochemical results could be assessed by toxicity scale while clinical adverse events were only described. Best response to treatment was determined by two authors (FAA and MEB) who retrospectively reviewed CT scans and used Response Evaluation Criteria in Solid Tumors (RECIST) v.1.1 to categorize the response as either complete remission (CR), partial remission (PR), stable disease (SD) or progressive disease (PD) [23].

### Statistical analysis

Statistical analyses were performed using SPSS 20 (IBM, Armonk, NY, USA). Data were expressed as median with the interquartile range (IQR) and percentage, unless otherwise specified. Survival analyses were conducted by Kaplan-Meier method and compared with log-rank test. PFS was calculated from the date of first dose of trabectedin to the date of disease progression as documented on CT scan. Patients who only received one cycle of trabectedin or died before their first on-treatment CT scan were excluded from PFS analysis. Patients were censored at time of death or last follow-up at MSH/PM, whichever occurred first. Overall survival (OS) was calculated from the date of first dose of trabectedin to the date of death or last follow-up at MSH/PM, whichever occurred first. The cut-off date for follow-up in this study was March 31, 2015. Statistical significance was set at *p*-value <0.05.

## Results

### Patient and tumor characteristics

A total of 77 patients were treated with trabectedin for unresectable advanced or metastatic STS (Table 1). Patients had a median age of 52y (IQR: 45–61y) and were predominately female (62.3 %). Overall patients had a good performance status before treatment (97.4 %). The majority of patients (57.1 %) had at least one comorbidity of which hypertension (*n* = 13), hypothyroidism (*n* = 7), diabetes mellitus (*n* = 6), depression (*n* = 6), and smoking (*n* = 6) were the most common. Nine patients (11.7 %) had a prior history of cancer including bladder (*n* = 3), breast (*n* = 2), thyroid (*n* = 2), and lymphoma (*n* = 2).

**Table 1:** Patient and tumour characteristics at time of starting trabectedin

Variable	N (%)
Age (years), median (IQR)	52 (45–61)
Gender	
Male	29 (37.7)
Female	48 (62.3)
ECOG performance status	
0	32 (41.6)
1	43 (55.8)
2	2 (2.6)
Number of comorbidities	
0	33 (42.9)
1	19 (24.7)
≥ 2	25 (32.4)
Prior history of cancer	
No	68 (88.3)
Yes	9 (11.7)
Histology	
Leiomyosarcoma	32 (41.6)
Others	21 (27.2)
Liposarcoma	14 (18.2)
Synovial sarcoma	10 (13)
Grade	
I	8 (10.4)
II	16 (20.8)
III	27 (35.1)
Not graded/recorded	28 (36.4)
Site of primary tumour	
Thorax	6 (7.8)
Abdomen/pelvis	11 (14.3)
Retroperitoneum	13 (16.9)
Uterus	21 (27.3)
Extremity	26 (33.8)
Site of local recurrence (*n* = 20)	
Thorax	4 (20)
Abdomen/pelvis	12 (50)
Retroperitoneum	3 (15)
Extremity	1 (5)
Site of metastasis (*n* = 57)	
Brain	1 (1.8)
Lung	46 (80.7)
Thorax	4 (7)
Liver	15 (26.3)
Abdomen/pelvis	13 (22.8)
Bone	14 (24.6)
Extent of tumour	
Inoperable primary tumour	13 (16.9)
Locally recurrent	13 (16.9)
Recurrent metastatic tumour	44 (57.1)
Locally recurrent and metastasis	7 (9.1)

*ECOG* Eastern Cooperative Oncology Group

The most common STSs included leiomyosarcoma (41.6 %), liposarcoma (18.2 %), and synovial sarcoma (13 %). Approximately 27 % of patients had a variety of rare histologies (“other sarcoma”), which included spindle cell sarcoma, fibrosarcoma, clear cell sarcoma, high-grade pleomorphic undifferentiated sarcoma and rhabdomyosarcoma. Of patients who had information available, the majority of tumors were high grade (55.1 %).

Tumors were primarily localized in in the torso (66.2 %). The most common sites of primary disease were uterus (27.3 %), retroperitoneum (16.9 %), and abdomen/pelvis (14.3 %). At the time of starting trabectedin 51 (66.2 %) patients had one or more metastasis. Patients had metastasis with the following number of sites involved: one (52.6 %), two (33.3 %), and three or more (14 %). Anatomical distribution of metastasis was as follows: lung (80.7 %), liver (26.3 %), abdomen/pelvis (22.8 %), and bone (24.6 %)

### Treatment before trabectedin

All 77 patients received first-line chemotherapy before staring trabectedin. The majority of patients (71.4 %) received at least two lines of chemotherapy before starting trabectedin. Prior to trabectedin, 11 patients underwent radiation therapy with the following intent: neoadjuvant (9.1 %), adjuvant (36.7 %), and palliative (54.5 %). A total of 64 (83.1 %) patients had surgery for their primary tumor prior to starting trabectedin while the remaining 13 patients had inoperable tumors. Margin status after surgery for patients’ primary STS was as follows: R0 (76.6 %), R1 (20.3 %), and R2 (3.1 %). Local recurrence was diagnosed in 20 (31.3 %) patients. Distribution of site of local recurrence was as follows: abdomen/pelvis (50 %), thorax (20 %), retroperitoneum (15 %), and extremity (5 %). Twenty-three patients underwent additional surgery including positive margin excision (17.4 %), local recurrence excision (17.4 %), and metastectomy (65.2 %). Trabectedin was provided for patients with recurrent metastatic (57.1 %), locally recurrent (16.9 %), inoperable primary (16.9 %), and both locally recurrent and metastatic (9.1 %) tumours.

### Trabectedin treatment characteristics

Median time from diagnosis to start of trabectedin was 22.4 months (m) (IQR: 13.3–44.9 m). Median number of cycles of trabectedin was 2 (range: 1-17) during a median time of 1.5 m (range: 0.3–16 m). Trabectedin was primarily provided as second- and third-line chemotherapy in 28.6 % and 44.2 % patients, respectively; while the remaining 27.3 % received it as ≥4-line treatment. Of the 77 patients, 2 patients were started on trabectedin after developing severe toxicities with other lines of chemotherapy while the remaining 75 patients received treatment due to disease progression.

**Fig. 1:**
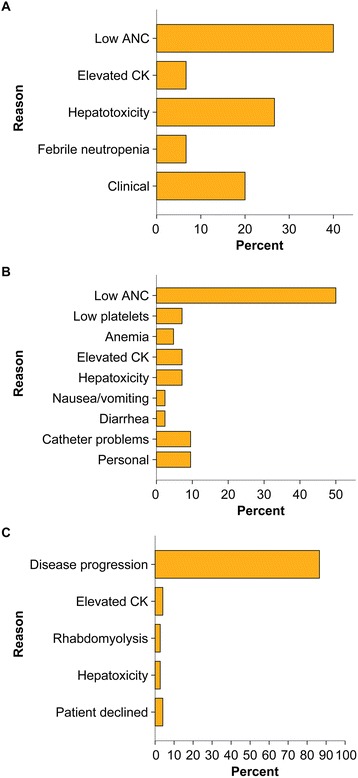
Trabectedin dose and schedule modifications. **a** Reasons for dose reductions (*n* = 15 patients, 19.5 %). **b** Reasons for schedule delay (*n* = 31, 40.3 %). Number of delayed cycles per patient: one (*n* = 25, 80.7 %), two (*n* = 5, 16.1 %) and three (*n* = 1, 3.2 %). **c** Reasons to discontinue trabectedin (*n* = 72, 93.5 %). Abbreviations: ANC, absolute neutrophil count; CK, creatine kinase

The majority of patients (96.1 %) started treatment at a dose of 1.5 mg/m^2^. Three patients started treatment at 1.2 mg/m^2^ because they were considered frail; two of these patients eventually had their dose increased to 1.5 mg/m^2^ because they tolerated treatment. Frequency and reasons for dosage and schedule modifications are depicted in Fig. 1. A total of 15 patients (19.5 %) required dose reductions primarily owing to low absolute neutrophil count (ANC) (40 %), followed by hepatotoxicity (26.7 %) and clinical reasons (20 %) (Fig. 1a). In the majority of cases, patients required a single dose reduction (86.7 %), but dosage was generally readjusted to normal (66.7 %). A total of 31 (40.3 %) patients had treatment delays primarily because of low ANC (61.3 %), catheter problems (12.9 %), and personal reasons (12.9 %) (Fig. 1b). Number of treatment delays per patient was as follows: one (80.6 %), two (16.1 %), and three (3.2 %). Trabectedin therapy was discontinued in 72 patients (93.5 %) because of disease progression (84.7 %), severe adverse events (9.7 %), and patient decision (5.6 %) (Fig. 1c). Currently five patients are undergoing treatment with trabectedin.

### Trabectedin-related toxicities

A total of 150 hematological and/or biochemical toxicities occurred in 60 patients (Table 2). The median number of hematological and/or biochemical toxicities per patient was 2 (IQR: 1-3). The most common toxicities included low ANC (29.3 %) and elevated liver enzymes (26 %). Events of severe toxicity (CTCAE grade ≥3), primarily occurred because of elevation of liver enzymes (18.7 %), low ANC (12.7 %), and elevated CK (3.3 %). A total of 25 clinical adverse events occurred with the following distribution: nausea/vomiting (*n* = 18), fatigue (*n* = 5), diarrhea (*n* = 1), and leg edema (*n* = 1).

**Table 2:** Trabectedin-related toxicities

Type of toxicity	Grade 1 n (%)	Grade 2 n (%)	Grade 3 n (%)	Grade 4 n (%)	Grade 5 n (%)
Hematological					
Anemia	8 (10.4)	3 (3.9)	6 (7.8)	-	-
Low ANC	1 (1.3)	24 (32.4)	15 (20.3)	4 (5.2)	-
Thrombocytopenia	5 (6.5)	-	1 (1.3)	2 (2.6)	-
Lymphoenia	1 (1.3)	-	1 (1.3)	-	-
Febrile neutropenia	-	-	2 (2.6)	-	-
Biochemical					
Elevated ALP	12 (15.6)	2 (2.6)	4 (5.2)	2 (2.6)	-
Elevated GGT	10 (12.9)	2 (2.6)	5 (6.5)	2 (2.6)	-
Elevated CK	4 (5.2)	1 (1.3)	1 (1.3)	2 (2.6)	2 (2.6)
Elevated AST	3 (3.9)	2 (2.6)	5 (6.5)	2 (2.6)	-
Elevated ALT	6 (7.8)	1 (1.3)	5 (6.5)	3 (3.9)	-
Elevated bilirubin	-	-	1 (1.3)	-	-

*ALP* alkaline phosphatase, *ALT* alanine transaminase, *ANC* absolute neutrophil count, *AST* aspartate aminotransferase, *CK* creatine kinase, *GGT* gamma-glutamyl transferase

Deaths attributed to drug-related events were reported in two patients both of which were due to rhabdomyolysis. One patient with recurrent metastatic poorly differentiated leiomyosarcoma in the abdomen developed elevated CK after two cycles. The patient was admitted for rhabdomyolysis and treated, but died from acute tubular necrosis. Another patient with an inoperable retroperitoneal grade III malignant fibrous histiocytoma died after three cycles of trabectedin. The patient presented with severe bilateral lower limb pain and edema and blood work suggested ongoing rhabdomyolysis and acute renal failure. Despite treatment, the CK continued to increase, reaching 18,400U/L, until the patient eventually developed anuria and died secondary to acute renal failure.

### Effectiveness

**Fig. 2:**
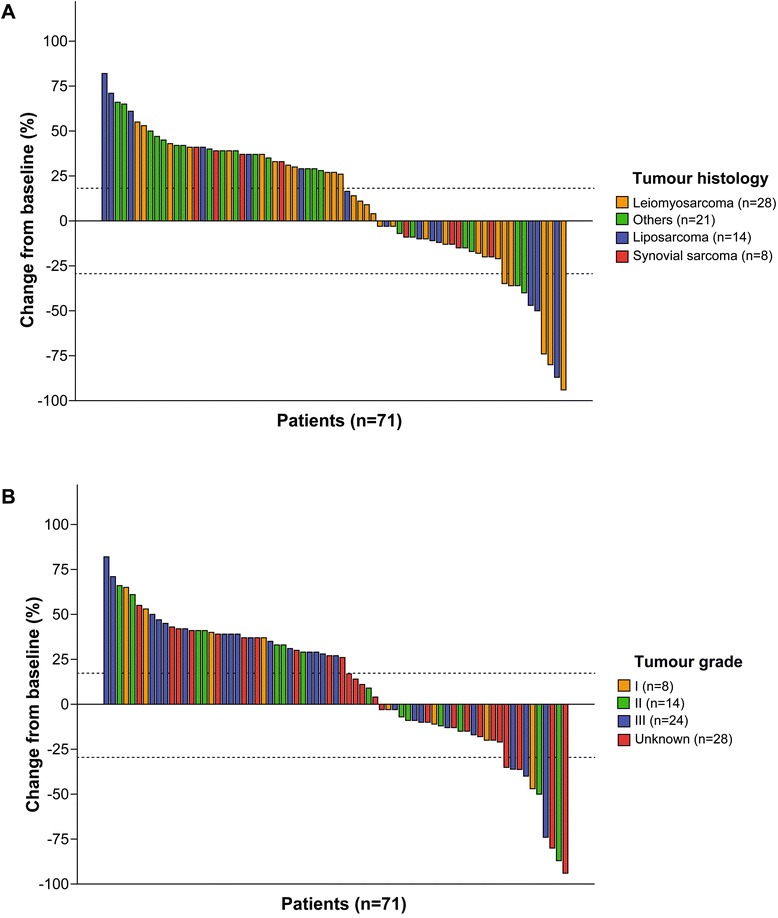
Best response to trabectedin in 71 patients with soft tissue sarcoma. Waterfall plots depict change from baseline in sum of longest diameters of target lesions for each patient according to tumor histology (**a**) and grade (**b**). Six patients were excluded from this analysis because CTs were not performed: 3 stopped treatment after cycle 1 for personal reasons, 1 recently started treatment and 2 had early toxicities requiring treatment suspension. Cut-off levels were based on Response Evaluation Criteria in Solid Tumors (RECIST) definitions [23]

**Fig. 3:**
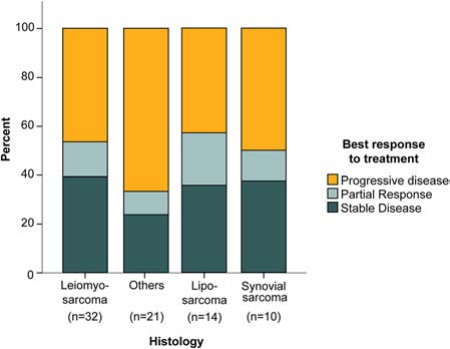
Distribution of best type of response to trabectedin according to tumor histology. Best response was assessed using Response Evaluation Criteria in Solid Tumors (RECIST) (*n* = 71)

**Fig. 4:**
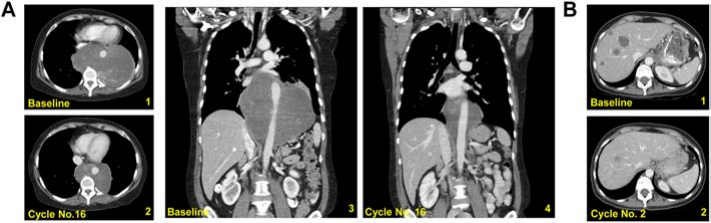
CT scans of patients who responded to trabectedin. **a** Patient with recurrent metastatic grade II myxoid liposarcoma encasing the aorta. Paired axial (1,2) and coronal (3,4) cuts showing a partial response after 16 cycles of trabectedin. **b** Patient with recurrent poorly differentiated leiomyosarcoma that metastasized to the liver. Paired axial (1,2) cuts showing a partial response after 2 cycles of trabectedin

Seventy-one were assessed for effectiveness. Six patients were excluded because they did not have on-treatment CT scans at the study cut-off date: three patients stopped treatment after cycle 1 for personal reasons, one patient had only one cycle, and two patients had early, severe toxicities requiring treatment suspension. Figure 2 illustrates best response to trabectedin as measured by RECIST. While CR was not observed in any patient, partial PR and SD were recorded as best response in 10 (14.1 %) and 24 (33.8 %) patients, respectively. The remaining 37 (52.1 %) patients showed PD. Figure 3 depicts the distribution of best type of response to trabectedin according to tumor histology. Trabectedin tended to induce PR in patients with liposarcoma (21.4 %) and leiomyosarcoma (12.5 %) (Fig. 4).

**Fig. 5:**
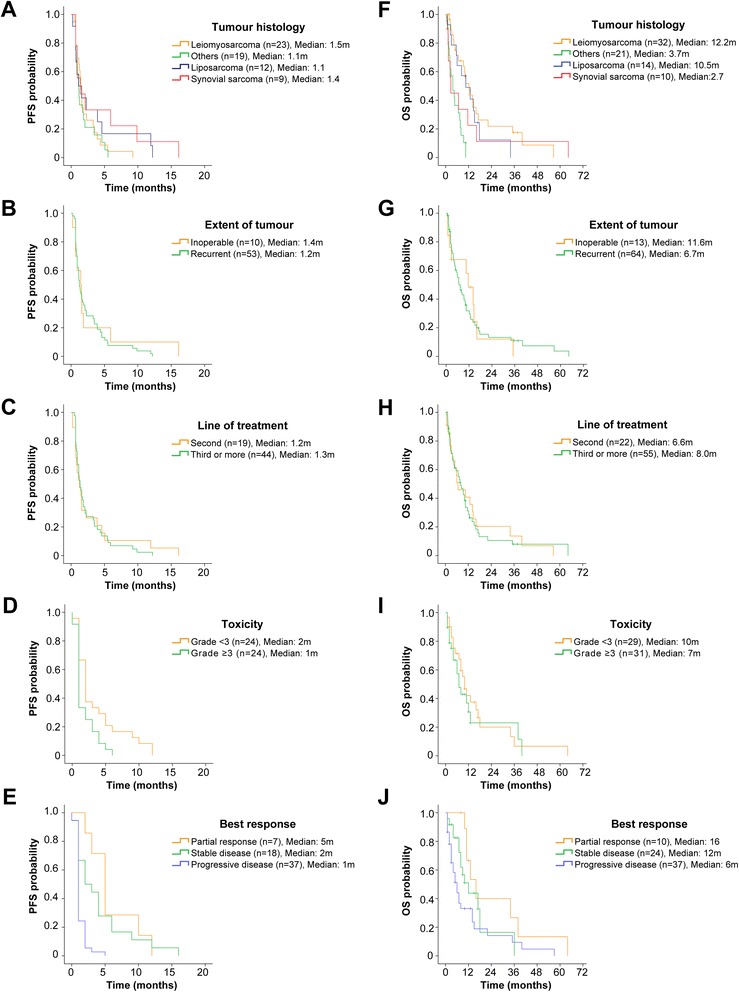
Trabectedin survival analyses. Kaplan-Meier (KM) survival curves for progression-free survival (PFS) (*left column*) and overall survival (OS) (*right column*) stratified by tumor histology (**a**, **f**), extent of tumor at presentation (**b**, **g**), line of treatment with trabectedin (**c**, **h**), severity of toxicity (**d**, **i**) and best response by RECIST (**e**, **j**). Abbreviations: m, months; OS, overall survival; PFS, progression free survival, RECIST, Response Evaluation Criteria in Solid Tumors

The median PFS was 1.3 m (IQR: 0.7–3.5 m). Fig. 5a-e depicts PFS stratified by factors with potential impact on outcome. PFS was significantly higher in patients who had grade <3 toxicities relative to those with grade ≥3 toxicities (1 m versus 2 m, *p* = 0.02). PFS was also significantly higher in patients who had PR or SD relative to those with PD (PR: 5 m versus PD: 1 m, *p* < 0.0001 and SD: 2 m versus PD: 1 m, *p* < 0.0001). Trabectedin did not induce significant improvements in PFS depending on histology, extent of tumor at presentation, or current number of line of chemotherapy. The median follow-up time was 6.6 m (IQR: 2.3–12.7 m). The median OS for this cohort was 6.7 m (IQR: 2.3-12.7 m). Fig. 5f-j depicts OS stratified by factors with potential impact on outcome. Patients with leiomyosarcoma or liposarcoma had significantly higher OS relative to other types (leioymyosarcoma: 12.2 m versus others: 3.7 m, *p* < 0.0001 and liposarcoma: 10.5 m versus others: 3.7 m, *p* = 0.002). OS was significantly higher in patients who had PR relative to those with PD (PR: 16 m versus PD: 6 m, *p* = 0.003). OS did not improve depending on extent of tumor at presentation, current number of line of chemotherapy, or severity of toxicity. At the end of the follow-up period, 54 (70.1 %) patients had died of their disease (*n* = 52) or trabectedin-related causes (*n* = 2). Twenty-three patients are alive and undergoing the following treatments: other chemotherapies (*n* = 9), palliative care (*n* = 6), trabectedin (*n* = 5), and targeted therapies (*n* = 3).

## Discussion

This study assessed the safety and effectiveness of trabectedin in a ‘real-world’ setting in patients with advanced STS. Between 2005 and 2014, our high-volume adult sarcoma centre treated 77 patients with trabectedin, the largest retrospectively published cohort of patients in North America to date. Our cohort resembles that of other larger studies in terms of patient and tumor characteristics [13, 24, 25]. Our results confirm that trabectedin is a well-tolerated agent that appears to induce some response in patients with advanced STS who previously failed standard-of-care chemotherapy. Trabectedin has a manageable safety profile with common toxicities including reversible low ANC, anemia, thrombocytopenia and hepatotoxicity and rare, severe clinical consequences such as elevated CK and rhabdomyolysis. Cumulative toxicities were not observed. At best, trabectedin induced a PR, particularly in patients with lipo- and leiomyosarcoma. Patients who had grade ≥3 toxicities and PD had significantly worse PFS relative to their counterparts. OS was significantly higher in patients with leiomyo- or liposarcoma relative to their counterparts. Given that our treatment inclusion criteria were less restrictive than those of clinical studies, our study depicts how trabectedin acts in a real clinical setting.

Trabectedin treatment in our cohort was reasonably well tolerated with an overall safety profile consistent with that of previous studies. As previously reported, the most common toxicities included self-limiting low ANC and elevated liver enzymes [25, 26]. Neutropenia was the most common drug-related toxicity and its incidence and severity is particularly higher with the dosing schedule used by our group [25]. Neutropenia followed a predictable and reversible course and was rarely associated with fever (1.9 %) or infection (1.8 %) as also noted in our cohort [25, 27]. Elevated liver enzymes occurred mainly in the first weeks of the first cycle and levels generally returned to baseline by day one of cycle two [25, 28]. Excluding patients with a known history of active liver disease and closely monitoring patients provides an adequate opportunity to adjust treatment. Notably, the incidence of bilirubin and alkaline phosphatase elevations was low and was not cumulative as previously reported [25].

In this study, the prevalence of grade ≥3 elevated liver enzymes and low ANC was within the rates reported in previous studies (low ANC: 33–61 % and transaminitis: 20–57 %) [12–15, 29, 30]. Despite the frequency of grade ≥3 toxicities, they only accounted for 2.8 % of the reasons why trabectedin was discontinued. Additionally events requiring in-hospital management affected 10 % of our patients, which is similar to the rate reported by other groups (9.4–17 %) [16, 24]. Clinical manifestations of severe hepatic injury are rare; therefore the changes observed in liver function tests mainly represent biochemical changes without permanent hepatic injury [25]. Post-treatment liver biopsies showed no evidence of persistent liver histopathological changes attributable to trabectedin [31].

Clinical adverse events were common in our patients. At least 20 % of patients develop clinical symptoms including nausea (64.7 %), fatigue (58.3 %), and vomiting (40.1 %) [25]. Nausea and vomiting, often associated with trabectedin, can be mitigated by pre-treating patients with dexamethasone [32]. Although the mechanism is yet unclear, the protection by dexamethasone against trabectedin-mediated toxicity may be attributed to enhanced Mrp2 biliary excretion and increased metabolism by CYP3A1/2 [33]. Notably, the adverse events commonly induced by cytotoxic chemotherapy or that are potentially dose-limiting, debilitating, cumulative and/ or life threatening are rare with trabectedin [25]. Overall trabectedin’s safety profile compares favorably with that of current standard-of-care agents used against STS [34].

Dosage and scheduling adjustments were in concordance with those reported by other studies. Dose reduction was necessary in 19.5 % of patients, which is well within the rate reported in the literature (14–48 %) [13, 17, 24, 26]. As expected the primarily causes for dose reduction were either low ANC or elevated liver enzymes [13, 26]. Approximately 40 % of our patients required treatment delays. Other studies have reported lower rates (27.7–36 %), but continue to find that neutropenia and increased transaminases are the two main causes [13, 17, 25]. Treatment discontinuations due to toxicity was necessary in 9.7 % patients, which is similar to the rate reported in the literature (8–10.2 %) [17, 25]. The primary reasons for treatment discontinuance include disease progression (63 %) [17].

Deaths attributed to drug-related events occurred in two patients and were attributed to rhabdomyolysis. The reported death rate is 0.5–1.7 % [17, 24, 25]. Deaths generally occur during the first two cycles of treatment and are mainly due to rhabdomyolysis [14, 25, 35]. Less frequently trabectedin causes death by inducing severe myelosuppression and respiratory failure [17]. Periodic monitoring of creatine phosphokinase as well as awareness of clinical manifestations is recommended for timely intervention.

Twenty-three percent of patients in our study received ≥6 cycles. In other studies a slightly higher percentage of patients (30–34 %) received an equivalent number of cycles of treatment suggesting an acceptable toxicity profile that allows prolonged treatment in certain patients [16, 24]. The number of patients undergoing long-term treatment would have been higher had they not progressed as trabectedin lacks cumulative toxicities [16, 24]. In a study that grouped data from 11 French centres, Blay *et al* reported that among 56 patients who were not progressing after six cycles, the 40 who continued treatment had a significantly higher PFS and OS relative to other patients [16]. Certainly these results must be taken in context of the retrospective nature of that study nevertheless maintenance therapy in patients with advanced STS is an option worth evaluating.

In our study both the median PFS and OS were on the lower end of what has been previously reported (PFS: 1.7–3.4 m and OS: 8.9–15.8 m) [12, 14, 15, 18, 36]. Our lower survival outcomes may be due to the fact that trabectedin was primarily given as a third- or more line of treatment in the majority of our patients. Other studies have reported higher survival outcomes because patients were not as heavily pre-treated as our cohort. In a study by Le Cesne *et al* in which only 58.7 % of 885 patients received trabectedin as a third- or more line of treatment, the median PFS and OS were 4.4 m and 12.2 m, respectively [24]. In another study where 32 % of the cohort received trabectedin as a third or more line of treatment, the median PFS and OS were 3.7 and 8.8 m, respectively [37].

Trabectedin has the potential to provide clinically meaningful benefits to a specific subset of STS patients who have failed standard-of-care treatment particularly if their tumours are either leiomyo- or liposarcoma. Both these subtypes of STS had an OS that was significantly higher relative to patients with other types of STS. This finding was previously shown in other studies in which the median OS was 12-16 m [13, 14, 17]. The severity of toxicities appeared to have an effect on PFS, but not OS as previously shown [16]. A possible explanation is that patients who develop severe toxicities after trabectedin receive a lower number of cycles because trabectedin is discontinued therefore decreasing their chances of responding to treatment. Objective radiological response to treatment as measured by RECIST was also associated with improved OS. Despite the fact that trabectedin induced a moderate radiological response (PR and SD) of 48 %, as previously reported [17, 36], the effect on tumor burden was enough to significantly improve PFS and OS. In another study patients who responded to trabectedin (PR or SD) also had a significantly higher PFS (7.7 m versus 2.1 m, *p* < 0.0001) and OS (12.1 m versus 5.5 m, *p* = 0.01) [37].

## Conclusion

Trabectedin has an acceptable and manageable safety profile and provides encouraging anti-tumor activity particularly in patients with leiomyo- or liposarcoma who failed standard-of-care agents. Trabectedin does not develop cumulative toxicity even in patients who receive a high number of cycles. Response to treatment according to RECIST criteria was modest, especially in patients with lipo- and leiomyosarcoma. OS was significantly improved in patients with leiomyo- or liposarcoma relative to other types of STS. The clinically meaningful benefits provided by trabectedin are comparable to those previously observed in clinical trials and other real-world case series. Our data further support the benefits of trabectedin in patients with advanced leiomyo- and liposarcoma who have failed standard-of-care agents.

## Abbreviations

ANC: absolute neutrophil count
CR: complete remission
CTCAE: common terminology criteria for adverse events
ECOG: Eastern Cooperative Oncology Group
IQR: interquartile range
M: months
OS: overall survival
PD: progressive disease
PFS: progression-free survival
PR: partial remission
RECSIT: response evaluation criteria in solid tumors
SD: stable disease
STS: soft tissue sarcoma
TTP: time to progression
Y: years old
